# Identification of Myotropic Neuropeptides from the Brain and Corpus Cardiacum-Corpus Allatum Complex of the Beetle, *Zophobas atratus*

**DOI:** 10.1673/031.010.14116

**Published:** 2010-09-17

**Authors:** Pawel Marciniak, Neil Audsley, Mariola Kuczer, Grzegorz Rosinski

**Affiliations:** ^1^ Department of Animal Physiology and Development, Adam Mickiewicz University, Poznań, Poland; ^2^ The Food and Environment Research Agency, Sand Hutton, York, United Kingdom; ^3^ Institute of Chemistry, Wroclaw University, Wroclaw, Poland

**Keywords:** peptidomics, MALDI-TOF, insects, beetles, pyrokinin, sulfakinin, myosuppressin

## Abstract

The neuropeptide profiles of the two major neuro-endocrinological organs, brain and retrocerebral complex *corpus cardiacum-corpus allatum* (CC/CA) of adult beetles, *Zophobas atratus* Fabricius (Coleoptera:Tenebrionidae) were analyzed by a combination of high performance liquid chromatography (HPLC) and matrix-assisted laser desorption ionization time of flight tandem mass spectrometry (MALDI TOF/TOF MS). The homological semi-isolated heart bioassay was used to screen HPLC fractions for myotropic activity in tissues, revealing several cardiostimulatory and cardioinhibitory factors from both the brain and CC/CA. Analysis of HPLC fractions by MALDI-TOF MS identified seven mass ions that could be assigned to other known peptides: leucomyosuppressin (LMS), *Tribolium castaneum* pyrokinin 2, sulfakinin 1, myoinhibitory peptide 4, a truncated NVP-like peptide, *Tenebrio molitor* AKH and crustacean cardioactive peptide. In addition, two novel peptides, myosuppressin (pEDVEHVFLRFa), which differs from LMS by one amino acid (E for D at position 4) and pyrokinin-like peptide (LPHYTPRLa) were also identified. To establish cardioactive properties of some of the identified peptides, chemical synthesis was carried out and their activities were tested using the heart bioassay.

## Introduction

In insects, as in many other invertebrates, and vertebrates, neuropeptides are important messenger molecules that influence developmental, reproductive and behavioural processes (De Loof 2008). In the past few years, a large number of new neuropeptides have been identified from cockroaches (Neupert and Predel 2005), locusts (Baggerman et al. 2003), flies (Schoofs and Baggerman 2003), moths (Audsley and Weaver 2003), the honeybee (Audsley and Weaver 2006) and stink bugs (Predel et al. 2008). Recently, the release of insect genomes (from *Drosophila melanogaster, Anopheles gambiae, Aedes aegypti, Bombyx mori, Apis mellifera* and *Tribolium castaneum*), combined with very sensitive techniques, such as mass spectrometry, has provided powerful tools for studying insect peptidomes.

The release of the genome sequence of the red flour beetle, *T. castaneum* (BeetleBase website: www.bioinformatics.ksu.edu/BeetleBase), has resulted in the identification of over eighty neuropeptides and protein hormones from this insect (Li et al. 2008). A variety of neuropeptides have also been identified from *corpus cardiacum—corpus allatum* or head extracts of the yellow mealworm beetle, *Tenebrio molitor*, including the adipokinetic/hypertrehalosaemic peptides (Gäde and Rosinski, 1990), diuretic (Furuya et al. 1995; Furuya et al. 1998) and antidiuretic factors (Eingerheer et al. 2002, 2003) and the crustacean cardioactive peptide (CCAP) (Furuya et al. 1993). Recently, three pyrokinins Tenmo-PK 1 (HVVNFTPRLa), Tenmo-PK 2 (SPPFAPRLa), Tenmo-PK 3 (HL/ISPFSPRLa) and a myosuppressin (pEDVDHVFLRFa), that is identical to leucomyosuppressin (LMS) from the cockroach *Leucophaea maderae* (Holman et al. 1986a) have been identified in CC/CA and brain of *T. molitor* (Weaver and Audsley 2008).

Several myotropic peptide hormones have also been identified in the Colorado potato beetle, *Leptinotarsa decemlineata:* the oviduct motility stimulating peptide Led-OVM (Spittaels et al. 1991), a myotropic factor, Led-MNP-I (Spittaels et al. 1995a), proctolin and its bioanalogue Ala1-proctolin (Spittaels et al. 1995b), Led-NPF-1 and Led-NPF-2 from the short neuropeptide F family (Spittaels et al. 1996a) and a peptide identical to *Locusta migratoria* accessory gland LomAG-MT-I (Spittaels et al. 1996b). Furthermore, three pyrokinins, a pyrokinin-like peptide and a putative antidiuretic factor were isolated from the brain of *L. decemlineata* adults (Huybrechts et al. 2004; Lavigne et al. 2001). A peptide involved in reproduction control, designated Led-MAPG from the male accessory gland was also isolated from *L. decemlineata* (Schooneveld et al. 1997).

Adipokinetic/hypertrehalosaemic peptides have been isolated from several beetle species, including *Tribolium brevicordis* (Gäde et al. 2008), *Trichostetha fascicularis* (Gäde et al. 2006), *Melolontha melolontha, Geotrupes stercorosus* (Gäde 1991) and onitine beetles (Gäde 1997).

The present study has been undertaken to provide a more detailed insight into the endogenous (cardioactive) peptides from the brain-corpora cardiaca-corpora allata neurosecretory system of *Zophobas atratus* Fabricius (Coleoptera:Tenebrionidae). utilizing matrix-assisted laser desorption ionization time of flight mass spectrometry (MALDI-TOF MS). This technique has been previously used for the peptide profiling of neuro-endocrine tissues of several insect species (Audsley and Weaver 2003, 2006; Baggerman et al. 2003, Predel et al. 2008). To date only one peptide hormone, AKH, has been characterized from *Z. atratus* (Gäde and Rosinski, 1990). This beetle has been used as a model organism in various physiological bioassay s (Gäde and Rosinski, 1990; Marciniak et al. 2008; Quennedy et al. 1995) and therefore to further our understanding of the regulation of the physiology in *Z. atratus*, and enable comparison with other beetles, there is a need to identify the neuropeptides in this insect.

## Materials and Methods

### Insects

*Z. atratus* adults were obtained from a culture maintained at the Department of Animal Physiology and Development according to the Quennedy procedure (1995).

### Tissue extraction and liquid chromatography

A hundred brains and corpora cardiaca-corpora allata complexes were separately dissected from *Z. atratus* adults and placed into Eppendorf tubes containing 1 ml ice-cold 100% methanol. Tissues were infused at room temperature for 15–30 min and then removed. Tissue extracts were diluted with ten volumes of 0.1% trifluoroacetic acid for separation by reversed-phase high performance liquid chromatography (RP-HPLC). Separations were performed using a Beckman System Gold chromatographic system (Beckman Coulter Ltd., www.beckmancoulter.com), comprising a dual pump programmable solvent module 126 and a System Gold UV detector module 166. Samples were loaded via a Rheodyne loop injector onto a Jupiter C18 10 µm 300 Å narrow bore column (250 mm × 2.1 mm; Phenomenex, www.phenomenex.com) fitted with a guard column (30 mm × 2.1 mm) of similar packing material. The column was eluted with a linear gradient of 5–60% acetonitrile/0.1% trifluoroacetic acid, over 60 min at a flow rate of 0.2 ml/min, and elution was monitored at 214 nm. Fractions (1 min, 0.2 ml) were collected and concentrated to approximately 10 µl by centrifugal evaporation for mass analysis, or dried for bioassay.

### Heart bioassay

All HPLC fractions were re-suspended in 100 µl of *T. molitor* saline (274 mM NaCl, 19 mM KCl, 9 mM CaCl_2_, 5 mM glucose, and 5 mM HEPES, pH 7.0), to a concentration of 1 brain or CC/CA equivalent, and assayed *in vitro* using a semi-isolated *Z. atratus* heart prepared according to Gäde and Rosinski (1990). In the bioassay, the video microscopy technique and the computer-based method of data acquisition and analysis were used to study the action of the fractions on continuously perfused heart preparations as described previously (Marciniak et al. 2008). The activities of tested fractions are presented as percentage changes in the control frequency of the heart contraction. Proctolin (1×10^-9^ M), which stimulates contractions of the heart muscles in beetles (Rosinski, 1995), was used as a positive control.

### Mass analysis

Aliquots of HPLC fractions of the brain and CC/CA were analyzed by MALDI-TOF MS. The most abundant mass ions were subsequently fragmented for sequence analysis as described below.

Samples were diluted 1:1 with α-cyano-4-hydroxycinnamic acid (matrix) solution (Sigma-Aldrich) prepared at a concentration of 10 mg/ml in 50% acetonitrile/0.05% trifluoroacetic acid. One microlitre of sample/matrix was spotted onto the MALDI target plate and allowed to dry at room temperature.

Positive ion mass spectra were acquired in reflector mode using a Voyager-DE™ STR Biospectrometry workstation (Applied Biosystems, www.appliedbiosystems.com) or a Bruker Ultraflex II TOF/TOF MS (Bruker Daltonics GmbH, www.Bruker.com). The measured monoisotopic masses ([M+H]^+^) were compared to the monoisotopic masses of known peptides calculated using the Applied Biosystems Data Explorer software. Analyses by MALDI-post source decay (PSD) were performed on the Voyager workstation with angiotensin as standard. The fragmentation spectra were the accumulation of 7–8 spectral segments stitched together using the Applied Biosystems Data Explorer software. The MS/MS analyses using the Bruker Ultraflex utilised LIFT™ technology and data analysis by Flex Analysis software. Peptide sequences were determined using the Applied Biosystems software, or performed manually.

### Synthetic peptides

Four peptides identified in *Z. atratus* tissue extracts (pyrokinin 2, sulfakinin 1, myosuppressin 2 and NVP-like peptide were synthesized and tested for their effects on the heart of *Z. atratus.* Peptides were synthesized by the classical solid phase method according to the Fmoc-procedure (Fields and Nobel 1990). Amino acids were assembled either on a Wang (peptide acids) or Rink amide MBHA resin (peptide amides). As a coupling reagent HBTU in the presence of HOBt was used. The N-Fmoc group was removed with 20% piperidine in *N,N*-dimethylformamide (DMF). The peptide-resin was cleaved with trifluoroacetic acid in the presence of ethanedithiol (EDT). All peptides were purified by preparative HPLC on a Varian ProStar HPLC (www.varianinc.com), using a Tosoh Biosciences column (www.tosohbioscience.com), ODS-120T C18 (ODS 300 × 21.5 mm. Analytical HPLC was performed using a Thermo Scientific HPLC (www.thermoscientific.com) with a VYDAC C_18_ column (ODS 250 × 4.6 mm). The molecular weights of the peptides were determined with a Bruker Daltonics microTOF-Q mass spectrometer. Synthetic leucomyosuppressin was purchased from Bachem AG (www.bachem.com).

Synthetic peptides were dissolved in saline to yield a stock solution of 1 mM and were stored at -30 ^°^C. Working dilutions (1×10^-9^ M) were made from the stock solution in saline.

## Results

### Cardioactivity of HPLC fractions

**Figure 1.**
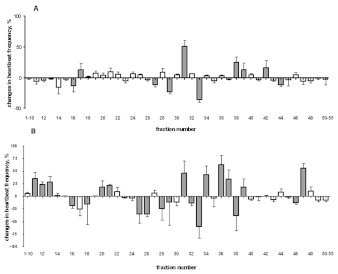
Cardioactive actions of HPLC fractions from methanol extracts of CC/CA (A) and brain (B) from *Zophobas atratus* adults. Activity is shown as a percentage change from controls (Ringer saline). Mean ± SEM, n = 3. Fractions different from control are highlighted in gray. High quality figures are available online.

**Figure 2.**
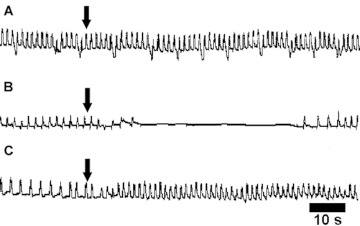
Myograms displaying contractile activities of the *Zophobas atratus* heart and the effects of (A) physiological saline, (B) fraction 33 from the CC/CA and (C) fraction 36 from the brain. Addition of saline or test compounds is indicated by the arrows. High quality figures are available online.

The *Z. atratus* heart rhythm remained regular during superfusion with saline and showed on average 40 ± 6 beats/minute. Myoactivity was widespread in HPLC fractions from both extracts of the CC/CA (Figure 4A) and the brain (Figure 1B). All active fractions caused fast and reversible changes in the heart contractile activity. From the CC/CA extract, ten HPLC fractions had activity on the *Z. atratus* myocardium and caused both chronotropic negative (fractions 16, 27, 29, 33, 44) and positive effects (fractions 17, 31, 38, 39, 42). Eleven HPLC fractions from the brain extract were cardiostimulatory (fractions 11, 12, 13, 20, 21, 31, 34, 36, 37, 39, 47) and a further ten were cardioinhibitory (fractions 16, 18, 25, 26, 28, 29, 32, 33, 38, 46). Figure 2 shows the myogram of the contractile activity of *Z. atratus* heart under control (Ringer saline) conditions and the effects of inhibitory and stimulatory factors from HPLC fractions. The addition of saline to *Z. atratus* heart had no effect on contractile activity (Figure 2A), whereas cardioinhibitory factors from the CC/CA (fraction 33; Figure 2B) and from the brain (fractions 33 and 38, not shown) induced short reversible cardiac arrests, which suggested occurrence of peptides with strong myoinhibitory properties. The cardiostimulatory effect of factors in fraction 36 from the brain is shown in Figure 2C.

### CC/CA and brain peptidomics

Analyses of HPLC fractions from both the CC/CA and the brain produced numerous mass ions, some of which had identical masses to known beetle peptides. The most abundant mass ions were fragmented for sequence analysis, although inadequate fragmentation of some peptides prevented sequence determination.

**Figure 3.**
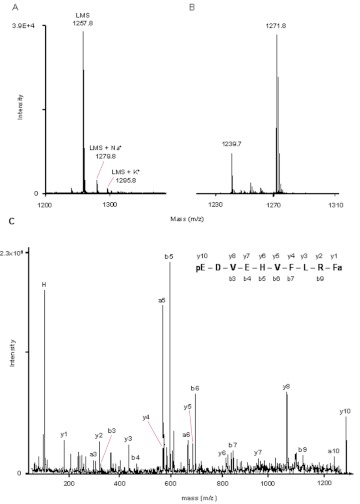
MALDI-TOF mass spectrum of HPLC fraction 33 (A) and fraction 36 (B) from a methanolic extract of adult *Zophobas atratus* CC/CA. Mass ions corresponding to leucomyosuppressin (LMS) are labeled. The fragment ion spectrum of the precursor ion (m/z) 1271.8 (from B) is shown in (C). The C-terminal and N-terminal ion signals are labeled. High quality figures are available online.

### Peptides associated with the CC/CA

One of the most prominent ion signals, with a monoisotopic mass ([M+H]^+^) of 1257.8 (fraction 33; Figure 3A; Table 1) corresponded to the calculated monoisotopic mass of leucomyosuppressin (1257.6; pEDVDHVFLRFa). Masses corresponding to the Na^+^ (1279.8) and K^+^ (1295.8) adducts of this peptide were also detected (Figure 3A). The fragmentation of the precursor ion m/z 1257.8 by MALDI-PSD produced C-terminal fragments (y ions) and N-terminal fragments (b ions), which were identical to those of synthetic LMS (not shown), confirmed the sequence of this peptide.

A putative second myosuppressin-like peptide (Zopat-MS-2), with a monoisotopic mass of 1271.8, was identified in fraction 36 (Fig. 3B). Fragmentation of this precursor ion produced y ions (y_1_, y_2_, y_3_, y_4_, y_5_, y_6_, y_7_, y_8_, y_10_), b ions (b_3_, b_4_, b_5_, b_6_, b_7_, b_9_) and a ions (a_3_, a_5_, a_6_, a_10_) in agreement with the sequence pEDVEHVFLRFa (Figure 3C).

**Figure 4.**
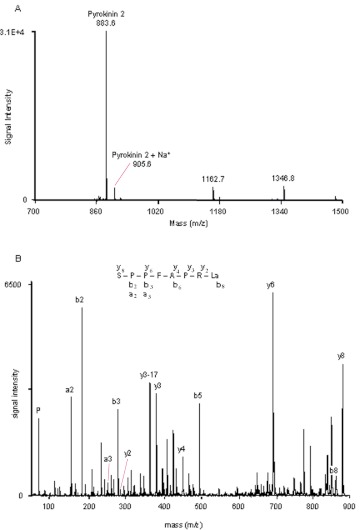
Mass spectrum of fraction 25 from HPLC fractionation of adult *Zophobas atratus* CC/CA (A). The mass ions associated with Trica-PK-2 are labeled. The fragmentation spectrum of precursor ion 883.6 (B), showing C-terminal and N-terminal fragments consistent with PK 2. High quality figures are available online.

The measured monoisotopic mass ([M+H]^+^) 883.6 in fraction 25 (Figure 4A) was consistent with the calculated monoisotopic mass of a coleopteran PRL-amide, pyrokinin 2 (883.5; SPPFAPRLa). The fragmentation of this precursor ion by MALDI-TOF/TOF produced five y ions (y_2_, y_3_, y_4_, y_6_, y_8_) together with four b (b_2_, b_3_, b_5_, b_8_) ions and two a (a_2_, a_3_) ions in agreement with this peptide (Figure 4B).

**Table 1.**
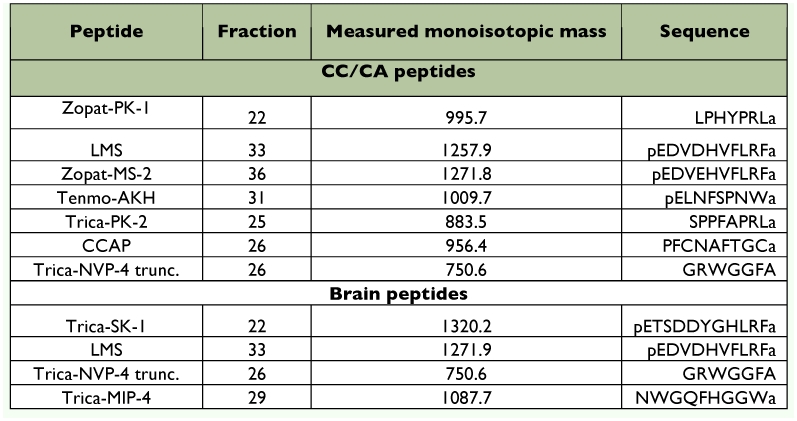
Peptides identified from the CC/CA and brain of *Z. atratus* by identical masses with known peptides and/or sequence analysis.

**Figure 5.**
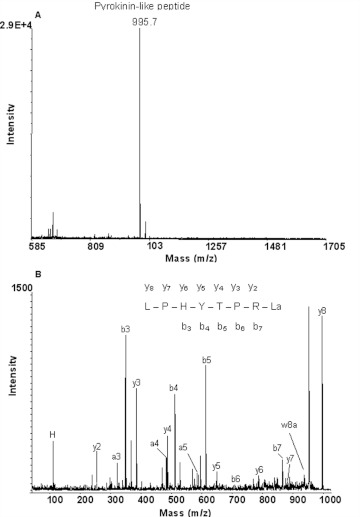
MALDI TOF mass spectrum of fraction 22 from the HPLC separation of adult *Z. atratus* CC/CA extract (A). The fragmentation spectrum of precursor ion (m/z) 995.7 (B) showing C-terminal and N-terminal fragments consistent with a novel peptide LPHYTPRLa. High quality figures are available online.

A novel pyrokinin-like peptide (Zopat-PK-like peptide), with a monoisotopic mass 995.7, was identified in fraction 22 (Figure 5A). Fragmentation of this ion produced almost all y ions (y2, y3, y4, y5, y6, y7, y8), five b ions (b3, b4, b5, b6, b7) and three a ions (a3, a4, a5) in agreement with the sequence LPHYTPRLa (Figure 5B). The presence of the w8a fragment at 936.3 confirms L as the N-terminal amino acid.

**Figure 6.**
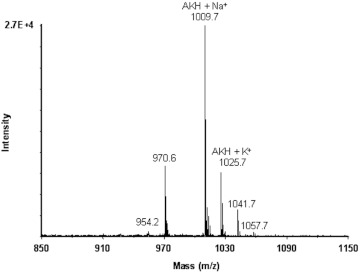
MALDI TOF mass spectrum of fraction 31 from the HPLC separation of adult *Z. atratus* CC/CA. The sodium and potassium adducts of AKH are labeled. High quality figures are available online.

Monoisotopic masses corresponding to the Na^+^ (1009.7) and K^+^ (1025.7) adducts of Tenmo-AKH (987.5; pEXNFSPNWa) were detected in fraction 31 (Figure 6). The sequence of this peptide was confirmed by MALDI-PSD (not shown).

The mass ions (m/z) 750.3 from fraction 26 corresponded to the truncated NVP-like peptide-4 (NVPL-4; GRWGGFA), and 956.4 to the crustacean cardioacceleratory peptide (CCAP; PFCNAFTGCa), but fragmentations of these mass ions were insufficient for sequence confirmation.

### Brain peptides

Monoisotopic mass in agreement with leucomyosuppressin (1257.6) in fraction 33 was also detected in HPLC fractions from methanol extracts of *Z. atratus* brains (Table 1). Analysis of mass ion 1257.6 by MS/MS produced the same fragmentation pattern as synthetic leucomyosuppressin confirming its sequence identity (results not shown).

**Figure 7.**
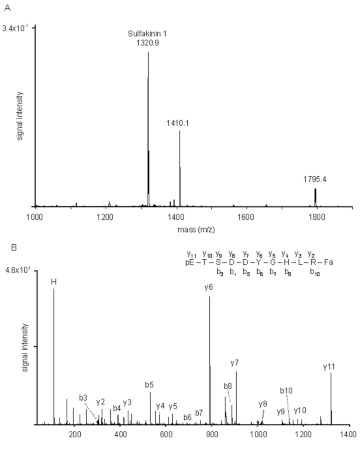
The mass spectrum of fraction 22 from the HPLC fractionation of a methanol brain extract from *Z. atratus* adults (A) is shown together with the fragmentation spectrum (B) of mass ion (m/z) 1320.9 (sulfakinin 1). The derived sequence is in agreement with this peptide (pETSDDYGHLRFa). High quality figures are available online.

The mass ion ([M+H]^+^) 1320.9 in fraction 22 (Figure 7A) corresponds to the monoisotropic mass of the coleopteran sulfakinin (1320.6; pETSDDYGHXRFa). The sequence of this peptide was confirmed by fragmentation by MALDI TOF MS/MS of the parent ion, which produced the full complement of y ions plus the b ions b_3_, b_4_, b_5_, b_6_, b_7_, b_8_ and b_10_ (Figure 7B).

The monoisotopic mass of 750.6 in fraction 26 (Table 1) is in agreement with the truncated Trica-NVP-4 (m/z 750.4; GRWGGFA). The fragmentation by MALDITOF MS/MS produced several y ions (y_2_, y_3_, y_4_, y_5_, y_6_, y_7_) and b ions (b_2_, b_4_, b_5_, b_6_, b_7_) (not shown).

The mass ion ([M+H]^+^) 1087.5 in fraction 29 (Table 1) corresponds to Trica-MIP-4 (NWGQFHGGWa) deduced from the *T. castaneum* precursor gene, but fragmentation by MS/MS was inadequate for sequence determination.

### Cardioactivity of synthetic peptides

**Figure 8.**
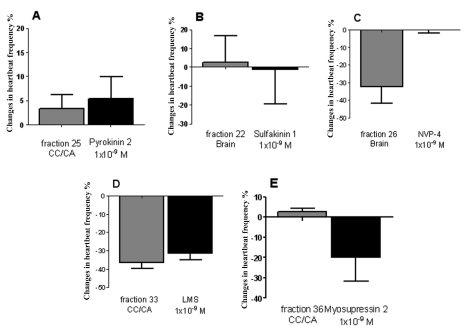
Cardioactivity of synthetic peptides Tenmo-PK 2 (A), Trica-SKI (B), Trica-NVP-4 trunc. (C), LMS (D) and Zopat-MS-2 (E) in comparison to their corresponding HPLC fractions (means ± SEM, n = 3–8). High quality figures are available online.

Figure 8 shows the effects of synthetic peptides and their corresponding HPLC fractions on *Z. atratus* heart. Pyrokinin 2 exerted a minor positive chronotropic effect at a concentration of 1×10^-9^ M (Figure 8A), similar to that of fraction 25 from the CC/CA. Neither sulfakinin 1 nor fraction 22 from the brain had any significant action on the myocardium (Figure 8B). In contrast, synthetic Trica-NVP-4 trunc had no effect on heart activity, whereas its corresponding HPLC fraction from the brain inhibited cardioactivity (Figure 8C). Heart contractile activity was inhibited 30% by 1×10^-9^ M synthetic LMS, in a similar manner to fraction 33 from the CC/CA (Figure 8D) and from the brain (Figure 1). A second synthetic myosuppressin (Zopat-MS-2) also had a negative chronotropic effect on the *Z. atratus* heart. This peptide was less potent then LMS, at a concentration of 1×10^-9^ M it inhibited heart contractions by 20% (Figure 8E). The corresponding HPLC fraction (36) was inactive in this bioassay (8E).

## Discussion

The neuropeptides associated with the brain and CC-CA of *Z. atratus* adults have been investigated by a combination of RP-HPLC and MALDI-TOF MS. Chromatography fractions were also tested on the heart bioassay to evaluate myotropic activity. By comparing measured molecular masses with calculated masses of known peptides from closely related beetles (*T. castaneum, T. molitor*) and MS/MS analysis we established amino acid sequences of seven peptide hormones. Except for AKH, these are the first neuropeptides isolated from the beetle *Z. atratus.*

The most prominent ion signal (1257.8) in both the brain and CC/CA corresponds to leucomyosuppressin, a member of the FMRFamide family first characterized from the cockroach *L. maderae* (Holman et al. 1986a). This peptide has subsequently been identified in other cockroaches, *Periplaneta americana, Blattella germanica* and *Diploptera punctata* (Predel et al. 2001, Aguilar et al. 2004, Bendena et al. 1997), and in the honey bee *A. mellifera* (Audsley and Weaver 2006) and beetles, *T. molitor* and *T. castaneum* (Weaver and Audsley 2008, Li et al. 2008), suggesting that this peptide is highly conserved in insects. Using antisera to FMRFamide, which most likely recognize the C-terminal RF-amide, and will cross-react with a wide range of FMRFamide like peptides (Orchard et al. 2001) FMRF-amide immunoreactivity was detected in the brain of *T. molitor* (Breidbach and Wegerhoff 1994). There has been no report of immunoactivity in the CC/CA complex of beetles, but in other insects, immunoreactivity to FMRFamides appears in both the CC and CA (Carroll et al. 1986; Rankin and Seymour 2001; Stay et al. 2003). Leucomyosuppressin has a strong myoinhibitory effect on visceral muscles in several insect species (Fuse and Orchard 1998; Matthews et al. 2008; Predel et al. 2001), and in the beetles *T. molitor* and *Z. atratus* this peptide causes dose-dependent reversible cardioinhibition (Skonieczna and Rosinski 2004). Chromatography fractions from the HPLC separation of methanolic extracts of both the CC/CA and the brain of *Z. atratus*, which contain this myosuppressin, also decrease the heart contractile activity and at high doses completely stop myocardium. A second, putative, myosuppressin-like peptide, designated Zopat-MS-2, with a predicted sequence of pEDVEHVFLRFa was also identified from the CC/CA. This peptide differs from leucomyosuppressin only by a conservative substitution of glutamic acid (E) for aspartic acid (D) at position 4. In other beetles (*Tribolium spp* and *T. molitor*) only one myosuppressin has been identified (Gäde et al. 2008; Li et al. 2008; Weaver and Audsley 2008) and the myosuppressin gene in *T. castaneum* encodes only one such peptide. This suggests that in *Z. atratus* either there may be two myosuppressin genes, or one gene encoding the two peptides. *Locusta migratoria* has two myosuppressins (Peeff et al. 1994), but it is also not known whether these peptides are encoded on the same or different genes. Alternatively the occurrence of Zopat-MS-2 could be the result of a point mutation in the gene resulting in the single amino acid substitution observed. Fraction 36, which contains this putative myosuppressin, had very little effect on heart contractions. The effects of Zopat-MS-2 may be masked by other (stimulatory) factors that co-elute with this peptide. Synthetic Zopat-MS-2 had similar effects on the heart as LMS, but was not as potent, suggesting the Substitution (E for D at position 4) may change the peptide's cardioinhibitory properties.

Pyrokinins (PKs), which were first identified from the cockroach, *L. maderae* (Holman et al. 1986b), are characterized by a FXPRLamide C-terminus. These peptides are pleiotropic as their effects on insects include the modulation of muscle contractile activity (Predel and Nachman 2001; Rosinski 1995). In beetles (*L. decemlineata, T. castaneum, T. brevicornis* and *T. molitor*), three pyrokinin (or neo-PBAN)-like peptides have been identified, or predicted from genome data (Huybrechts et al. 2004; Li et al. 2008; Weaver and Audsley 2008; Gäde et al. 2008), of which Trica-PK-2 (SPPFAPRLa) appears to be the most abundant. This peptide is also present in CC/CA of *Z. atratus*, but neither synthetic PK 2 nor the corresponding HPLC fraction (25) of CC/CA extract are active on the heart muscles. Similarly, the related PK from the cockroach *L. maderae* (Lem-PK; pETSFTPRLa) had very little stimulatory activity on the heart of *T. molitor* (Rosinski 1995). Pyrokinin 2 does however modify contractile activity of the hyperneural muscles, gut and oviduct of *P. americana* (Predel and Nachman 2001). A second pyrokinin-like peptide was identified from the CC/CA extract (fraction 22). This peptide (LPHYTPRLa), has a Y for F substitution, similar to that in the pyrokinin-like peptide identified from *L. decemlineata* (Huybrechts et al, 2004). Fragmentation using the Bruker Ultraflex LIFT™ technology produced the side chain cleavage necessary to distinguish between leucine and isoleucine, and hence assignment of L at the N-terminus by its w8a fragment (Nachman et al, 2005). No other PK-like peptide was identified in either tissue from *Z. atratus* Clearly, understanding the roles of these peptides in beetles requires further investigation.

Two sulfakinins Trica-SK-1 (pETSDDYGHLRFa) and Trica-SK-2 (GEEPFDDYGHMRFa) were predicted from the genome of *T. castaneum* (Weaver and Audsley 2008; Li et al. 2008). A peptide identical to Trica-SK1 was identified from the brain of *Z. atratus*, but no mass ion corresponding to Trica-SK2 was detected. The first insect sulfakinins were identified from the cockroach *L. maderae* by their ability to stimulate hindgut contractions (Nachman et al. 1986). They are also myotropic on the foregut and hindgut of *B. germanica* (Maestro et al. 2001), but have no myotropic activity on the gut of flies (Duve et al. 1994; Haselton et al. 2006). The effects of sulfakinins have not been tested on the gut of beetles, but synthetic sulfakinin 1 and its corresponding HPLC fraction (23) have no significant effects on the heart of *Z. atratus.* It is more likely that sulfakinins are involved in feeding regulation in beetles as described for other insects (Audsley and Weaver 2008). However, recent studies have shown that sulfakinins might have a possible role in heart activity regulation. Both sulfated and nonsulfated sulfakinins (DSK I, DSK II) induce cardiostimulatory effects in *D. melanogaster* dependent on the developmental stage (Nichols et al. 2009).

An AKH (Tenmo-AKH: pELNFSPNWa) had previously been identified from the CC of both *T. molitor* and *Z. atratus* (formerly *Z. rugipes*) (Gäde and Rosinski 1990), whereas two AKHs (pELNFSTDWa and pELNFTPNWa) are present in *T. castaneum* (Audsley and Weaver, 2008; Gäde et al 2008). From mass analysis of *Z. atratus* CC, only AKH was identified by the precursor ions 1009.7 and 1025.7, which represent the Na^+^ and K^+^ adducts of Temno-AKH, and its sequence was confirmed by MALDI-PSD. The protonated form of AKH is not usually detected using MALDI-TOF MS (Audsley and Weaver, 2003). Fraction 31 from the CC/CA (containing *Z. atratus* AKH) caused similar positive chronotropic effects on the beetle heart as synthetic AKH previously bioassayed by Rosiński (1995).

Six myoinhibitory peptides (MIPs) are predicted from the *T. castaneum* genome and/or were identified by direct analysis of nervous tissues by MALDI-TOF MS (Li et al. 2008; Weaver and Audsley 2008). However, only one mass ion in agreement with a known beetle MIP (MIP 4; NWGQFHGGWa) was measured in tissue (brain) extracts from *Z. atratus*, although its sequence was not confirmed due to insufficient fragmentation of the precursor ion. It is likely that other MIPs are present in *Z. atratus*, but their levels are either below the detection limits of the mass spectrometer, or their sequences (and hence masses) are not identical to the known beetle MIPs. To date, there are no reports of synthetic MIPs having been tested on the insect heart, but they are widely known as peptides with myoinhibitory activity (Schoofs et al. 1997). Fraction 29 from brain extract caused an inhibitory effect on beetle heart, but it is unclear whether this was due to a MIP, or other myoinhibitory factor.

Li *et al.* (2008) report that MS analysis of nervous tissue from *T. castaneum* identified seven NVP-like peptides of unknown function, which were not found in other tenebrionid beetles (Gäde et al. 2008). A mass ion (750.6) in agreement with a truncated form of Trica-NVP-4 (NVP-4 trunc; GRWGGFA) was measured in both HPLC fractions from *Z. atratus* CC and the brains. This sequence was confirmed by fragmentation of the parent ion from the brain. Synthetic NVP-4 trunc. had no effect on heart contractile activity, although its corresponding HPLC fraction inhibited heart contractions presumably due to a different, unidentified, peptide.

A mass ion (956.4) in agreement with CCAP was detected in the CC/CA of *Z. atratus*, but not the brain. No sequence confirmation was obtained due to the low abundance of this peptide, which may also explain lack of biological activity in the corresponding HPLC fraction. This peptide was originally isolated from the shore crab *Carcinus maenas* (Stangier et al. 1988) and has been identified in a variety of insects, including *T. molitor*, where it was shown to stimulate contractions of the myocardium (Furuya et al. 1993).

No other sequences related to existing insect peptide families have been established for mass ions that did not match masses of known beetle peptides, most likely due to insufficient fragmentation of the parent ion restricting complete sequence determination.

In conclusion, six neuropeptides (LMS, Zopat-MS-2, Trica-PK-2, Zopat-PK-like peptide, Trica-SK-1 and Trica-NVP-4 trunc.) have been identified from the brain and retrocerebral complex of *Z. atratus*, and the structure of its native AKH confirmed. In addition, two peptides (CCAP and Trica-MIP-4) have been assigned by their identical masses. It is clear from the heart bioassay data of HPLC fractions that both the CC/CA and the brain of *Z. atratus* contain many other (unidentified) myoactive compounds.
